# Inhibition of TNF-α Oncogene Expression by *Artemisia Annua* L. Extract Against Pioglitazone Side Effects in Male Albino Mice

**DOI:** 10.1007/s12033-023-00762-7

**Published:** 2023-05-13

**Authors:** Silvia Botrous, Ayaat Elmaghraby, Samar El-Achy, Yehia Mustafa, Effat Badr, Amany Haggag, Salah Abdel-Rahman

**Affiliations:** 1 Department of Genetics, Faculty of Agriculture, Alexandria University, Alexandria, Egypt; 2 Department of Nucleic Acid Research, Genetic Engineering and Biotechnology Research Institute, City of Scientific Research and Technological Applications, Alexandria, Egypt; 3 Department of Surgical Pathology, Faculty of Medicine, Alexandria University, Alexandria, Egypt

**Keywords:** Pioglitazone, Actos drug, *Artemisia annua*, Bladder cancer, TNF-*α* gene

## Abstract

Pioglitazone (Actos) is one of the most recent oral antidiabetic drugs for treating the second type of diabetes mellitus as a common chronic and lifelong disease, but with harmful side effects. The objective of this study is to evaluate the effectiveness of *Artemisia annua* L. extract against the Actos drug side effects in the male albino mice. In present study, the use of Actos drug alone induced hepatotoxicity, renal inflammation, hematological disorders and bladder cancer, which are manifested by biochemical abnormalities and histopathological changes, moreover, the severity of toxicity depends on its dose. In contrast, the concurrent treatment with both Actos drug (45 mg/kg) and Artemisia extract (4 g/kg) was effective against the harmful side effects of the Actos drug. Where, the biochemical, hematological and histopathological investigations showed that the hepatotoxicity, renal inflammation, hematological disorders and histopathological changes were improved using combination of Actos and Artemisia extract. In addition, the results of TNF-*ɑ* oncogene expression levels in bladder tissues were significantly decreased by about 99.99% using the mix of both Actos drug and Artemisia extract. In conclusion, these findings reveal that the *Artemisia annua* extract on TNF-*ɑ* oncogene expression level is very significant and effective natural product against harmful side effects of pioglitazone which associated with an increased risk of incident *bladder cancer* among *people*, but for application more studies must be achieved in that field.

## Introduction

Pioglitazone (Actos) is one of the most recent oral antidiabetic drugs for treating the second type of diabetes mellitus as a common chronic and lifelong disease which affects the ability of the body to use the energy found in the food [1]. Diabetes represents a major clinical and public health problem in the world, where, an estimated in 2016 referred that 1.6 million deaths were directly caused by diabetes and 2.2 million deaths were attributable to high blood glucose in 2012 [2]. Pioglitazone was originally authorized in the European Union in 2000 at doses of 15 or 30 mg once daily, but it was approved as an oral monotherapy for overweight diabetic patients and uncontrolled cases by diet and exercise in 2002 [3]. It is also considered a thiazolidinedione compound and *Peroxisome proliferator*-*activated receptor* (*PPAR*) *agonists* have emerged as promising repurposed drugs due to their anti-inflammatory activities. Its activity depends on the presence of insulin; however, it does not stimulate insulin secretion nor inhibit glucose absorption [4, 5]. Pioglitazone has additional multiple benefits, as an antiparkinsonian, antibacterial, hypolipidemic agent and influences on the learning, memory and oxidative stress, in addition to, glucose lowering effect [6]. In spite of its beneficial effects, there are emerging harmful issues regarding effects on congestive heart failure and bladder cancer [7]. There are many previous supportive studies about pioglitazone safety, but they do not provide conclusive evidence for the safety. Some studies indicated to an idiosyncratic hepatic reaction, liver failure and death due to the use of pioglitazone, while other studies referred to liver failure without associated deaths. Until now, no long-term studies are available about toxicity of pioglitazone repeated administration, whereas, its clinical use is currently under scrutiny because of the safety issues [8]. Where, the duration of use and the total dose over time of pioglitazone influenced the risk of bladder cancer [9]. Most of natural products, such as, medicinal plants and herbs confer protective effects against a wide range of cancers and side effects of drugs. *Artemisia annua* L. (AA) is a type of wormwood plant used in the treatment of malaria. The plant has been used in Chinese traditional medicine, where it is known as Qinghao, since at least the second century BC. Western medicine used the *Artemisia annua* plant in the 1970's after the increasing prevalence of drug-resistant Plasmodium parasites drove the Chinese government to research traditional remedies for the disease [10]. Since *Artemisia annua* was determined to have anti-malarial properties, the plant has been used primarily as a source of artemisinin, which is the major anti-malarial compound in the plant that has also been shown to have anti-proliferative effects on cancer specially breast cancer. As well as, some studies have been conducted to test its comparative effects on cancer based on the beneficial effects seen in the use of artemisinin and other compounds present in the plant in preventing cancer proliferation [11]. Tumor necrosis factor alpha (TNF-*α*) oncogene is a multifunctional cytokine that plays important roles in diverse cellular events such as cell survival, proliferation, differentiation, and death. As a pro-inflammatory cytokine, TNF is secreted by inflammatory cells, which may be involved in inflammation-associated carcinogenesis [12, 13]. *Artemisia annua* 90% ethanol Phyto-extract showed a greater ability to reduce TNF-*α* gene expression when used at the same dosage as artemisinin (ART). On the contrary, when the pro-inflammatory stimulus elicited by LPS was present, ART showed a greater anti-inflammatory response [14, 15]. The objective of the present study is to investigate therapeutic effect of *Artemisia annua* L. (AA) extract against the side effects of the Actos drug through the study of gene expression of TNF-*ɑ*. In addition, the assessment of histopathological examination of different organs (liver, kidney, testis and urinary bladder), biochemical analysis (liver profile test, kidney profile test and glucose level), and hematological analysis (complete blood count (CBC)) in treated and control animals (male albino mice).

## Materials and Methods

### Ethics

The study was performed in accordance with the guidelines for the care and use of laboratory animals approved by Institutional Animal Care and Use Committee AU-08-22-09-20-3-100 (Faculty of Medicine, Alexandria University, Egypt).

### Plant Extract Preparation

Artemisia (*Artemisia annua* L.) plants were obtained from local market in Alexandria, Egypt. For preparation of the extraction, different weights (1, 2 and 4 g) of the whole plants were put in dry cup and then 111 ml of boiled water (100 °C) was added (like tea preparation) and the whole extraction was left for 10 min and then filtered to get the drenched *Artemisia Annua* extract [16].

### Experimental Animal Model

Forty-five male albino mice were randomly selected from animal house at Faculty of Pharmacy, Pharos University, Alexandria, Egypt. The 45 male albino mice (Mus musculus/25-30 g) were transferred and housed at Medical Research Institute, Alexandria University. The animals were kept in plastic cages (three animals per cage) in a room maintained at proper environmental conditions: temperature 25 °C, humidity 50%- and 12-h light–dark cycle, then the animals were given free access food and water. The animals were daily observed for abnormal signs and finally, the animals were acclimatized for two weeks before starting the experiment. The whole experiment was divided into two parts (initial and main experiments). The initial experiment was classified to four groups (A, B, C and D): Group A is control, group B is Artemisia extract, group C is Actos drug and group D is Actos drug/Artemisia extract mix treatment (orally administered). Every group has three mice and the duration of this experiment was two weeks, as shown in (Table 1). According to the periods and the obtained results from the initial experiment, the main experiment was divided into two parts. One part was for 15 days and contains three groups (A, D1 and E), while the other part was for 30 days and contains the same three groups (A, D1 and E). Where, group A is control, group D1 is Actos/Artemisia mix and group E is a mix of Actos, Artemisia and Ferric.

**Table 1:** Treatment of experimental animal groups (A, B, C, D and E) with Artemisia extract and Actos drug

Group		Number of animals	Treatment (Dose dissolved in 111 ml distilled water/30g body weight/day)
*Initial experiment (15 days)*			
Group A (control)		3	Water + Food
Group B Artemisia extract (*Van der Kooy and Verpoorte, 2011*)	B1	3	1g
	B2	3	2g
	B3	3	4g
Group C Actos drug (*European medicines agency*)	C1	3	15mg
	C2	3	30mg
	C3	3	45mg
Group D Actos/Artemisia mix	D1	3	45mg/4g
	D2	3	45mg/4g (Actos after Artemisia with one hour)
*Main experiment (15 days)*			
Group A (control)		3	Water + Food
Group D1 Actos/Artemisia mix		3	45m g/4g
Group E Actos/Artemisia/Ferric mix		3	45m g/4 g/0.1ml
Main experiment (30 days)			
Group A (control)		3	Water + Food
Group D1 Actos/Artemisia mix		3	45m g/4g
Group E Actos/Artemisia/Ferric mix		3	45m g/4 g/0.1ml

### Biochemical and Hematological Analysis

Bio-clinical analysis of blood samples was tested for liver profile, kidney profile, glucose level and complete blood count. Liver profile contains two tests: Aspartate aminotransferase (AST) and Alanine aminotransferase (ALT), kidney profile contains two tests: urea, creatinine and glucose level (random blood sugar). Where, the blood samples for the previous tests were collected in serum-coated tubes (Cobas-C & Cobas- E (Roche) 26-instrument, USA). While in EDTA coated tubes (Swelab Alpha Basic, Sweden), blood samples were collected for complete blood count (CBC) which contains four parameters: Red blood cells (RBCs), leucocytes (WBC), platelets (PLT) and hemoglobin (HB). It should be noted that, the blood was centrifuged at 2000xg for 10 min to separate plasma and then stored at − 80 °C until analysis.

### Histopathological Studies

The different organs tissues (liver, kidney, testes and urinary bladder) from all the animal groups (A, B, C, D and E) were sliced and immediately fixed in 10% neutral buffered formalin for 24 h, washed with running water, dehydrated in ascending grades of alcohol (70%, 80%, 95% and absolute alcohol) and cleaned by immersion in xylene followed by impregnation in melted paraffin wax in oven at 60 °C for 1 h. The specimens were embedded in paraffin and were left to solidify at room temperature. Using a rotary microtone, sections of 5 µm thick of the previous tissues were cut and mounted on clean glass slides. With conventional hematoxylin and eosin (H&E) mix, these sections were stained for examination under the light microscope to find out any histopathological changes in these tissues. This part was carried out in Histopathology Department, Faculty of Medicine, Alexandria University, Egypt.

### Quantitative Real Time Polymerase Chain Reaction (qRT-PCR)

Total RNA from bladder mice’s tissue, was isolated using Gene JET RNA Purification Kit (Thermo Scientific, USA). The extracted RNAs from different samples were quantified and qualified (purity) using NanoDrop Spectrophotometer. Total RNAs samples were normalized (same concentration) to avoid any false increase in gene expression levels. Using SYBER Green 1-step qRT-PCR Kit (Thermo Scientific, USA), gene expression of TNF-ɑ (target gene) and *β*-actin (reference gene) were quantified by Real-Time PCR System (Thermo Scientific PikoReal) with the use of specific primers sequences (Forward/Reverse)forward 5′-CATCTTCTCAAAATTCGAGTGACAA-3′, reverse 5′-TGGGAGTAGACAAGGTACAACCC-3′ for TNF-*ɑ* oncogene [17]. and 5′-GCTGTATTCCCCTCCATCGT-3′/5′-GAGTCCTTCTGACCCATTCC-3’ for *β*-actin gene [18]. qRT-PCR was performed in a reaction mixture of 10 μl using 0.1 μl verso enzyme mix, 5 μl 1-step QPCR SYBER mix (1X), 0.5 μl RT-enhancer, 0.5 μl forward and reverse primers (10 pm), 0–2.9 μl water (PCR grade) and 0.5–3.4 μl RNA template (50 ng). qRT-PCR program was applied as one cycle of cDNA synthesis at 50 °C for 15 min, one cycle of Thermo-start enzyme activation at 95 °C for 15 min and followed by 40 cycles of denaturation at 95 °C for 15 s, annealing at 60 °C for 1 min and extension at 72 °C for 30 s.

### Statistical Analysis

Statistical analyses were performed using the statistical SPSS software (Version 13).

## Results

### Biochemical and Hematological Analysis

From the initial experiment and according to the biochemical and hematological analysis, 4 g was the best concentration of Artemisia extract in group B3, while 45 mg of the Actos drug was the best concentration in group C3. Where, the results of biochemical analysis of ALT, AST, urea, creatinine and glucose concentrations showed non-significant changes in all groups (B, C and D) compared to the control (group A), except one group (C3) was significant, as shown in Table 2. For the hematological analysis of HB, WBC, PLT and RBC counts, the results of HB and WBC showed non-significant changes in all groups (B, C and D) compared to the control group, except C3 group was significant. In contract, the results of PLT and RBC were non-significant in all groups. The main experiment was classified into two parts according to the duration (15 and 30 days). In 15 days of the main experiment, the results of biochemical analysis of ALT, AST, urea, creatinine and glucose concentrations showed non-significant changes in all groups (B3, D1 and E) compared to the control group, except one group (C3) was significant (Table 3). For the hematological analysis of HB, WBC, PLT and RBC counts, the results of HB and WBC showed non-significant changes in all groups (B3, D1 and E) compared to the control group, except C3 group was significant. In contract, the results of PLT and RBC were non-significant in all groups. In 30 days of the main experiment, the results of biochemical analysis activity of ALT, AST, urea, creatinine and glucose showed non-significant changes in all groups (B, D and E) compared to the control group, except one in group (C3) they were significant (Table 4). The results of hematological analysis of HB, WBC, PLT and RBC counts, showed that HB, WBC and PLT were non-significant changes in all groups (B, D and E) compared to the control (group A), except in C3 group they were significant. While in all groups, the result of RBC was non-significant.

**Table 2:** Biochemical and hematological analysis in the initial experiment

Test	Groups	A	B1	B2	B3	C1	C2	C3	D1	D2
*Biochemical analysis*										
ALT	M ± SD	66.72 ± 0.28	68.68 ± 0.25	70.66± 0.29	73.26 ± 0.29	503.2 ± 0.36	886.9± 0.27	1270.4±0.25	68.58±0.25	91.68±0.25
	Significance	0.967	0.956	0.913	0.913	0.369	0.136	0.016	0.956	0.956
AST	M ± SD	185.7± 0.25	188.74±0.21	189.6±0.3	193.4± 0.25	339.6± 0.25	829.6± 0.27	1319.6±0.32	186.8±0.6	175.6±0.34
	Significance	0.953	0.193	0.126	0.135	0.135	0.117	0.018	0.839	0.276
Urea	M ± SD	19.83± 0.21	21.60±0.25	22.78± 0.25	26.13 ± 0.21	35.58 ± 0.25	40.40 ±0.27	45.28±0.25	21.68±0.25	18.68±0.25
	Significance	0.967	0.198	0.197	0.237	0.497	0.273	0.101	0.197	0.197
Creatinine	M ± SD	0.47± 0.008	0.49±0.008	0.52± 0.008	0.54± 0.008	0.40± 0.008	0.64± 0.008	1.1±0.009	0.47±0.008	0.45±0.01
	Significance	0.33	0.296	0.461	0.149	0.171	0.213	0.011	0.192	0.85
Glucose	M ± SD	101.6 ± 0.32	93.75±0.36	101.0 ± 0.25	103.6 ±0.3	96.67± 0.38	103.1±0.3	103.2±0.29	102.2±0.25	92.66±0.3
	Significance	0.985	0.405	0.384	0.169	0.498	0.331	0.341	0.202	0.169
*Hematological analysis*										
HB	M ± SD	14.34± 0.32	14.15±0.3	13.88± 0.25	14.55± 0.32	13.04± 4.37	14.38± 0.25	10.18±0.25	12.97±0.27	14.56±0.31
	Significance	0.688	0.829	0.815	0.694	0.936	0.315	0.021	0.867	0.878
PLT	M ± SD	544.6± 0.35	548.6±0.25	549.7 ± 0.29	553.8 ± 0.32	572.6± 0.34	791.5 ± 0.62	1050.6±0.34	552.6±0.32	570.6±0.34
	Significance	0.142	0.325	0.205	0.21	0.078	0.901	0.078	0.219	0.832
RBC	M ± SD	9.49 ± 0.01	9.70±0.16	9.62 ± 0.21	9.94 ± 0.11	9.61 ±0.2	8.39± 0.08	7.12±0.18	9.29±0.16	10.19±0.14
	Significance	0.918	0.954	0.491	0.937	0.853	0.708	0.545	0.967	0.907
WBC	M ± SD	8.51± 0.17	8.24±0.19	8.54±0.3	8.92± 0.26	10.50± 0.35	13.26± 0.13	21.74±0.13	8.66±0.25	7.17±0.2
	Significance	0.168	0.956	0.171	0.129	0.897	0.661	0.014	0.028	0.481

Values are expressed as mean (M ±) and SD (Standard Deviation) with significant at *p* < 0.05

**Table 3:** Biochemical and hematological analysis in the main experiment (15 days)

Test	Groups	A	B3	C3	D1	E
*Biochemical analysis*						
ALT	M ± SD	44.79 ± 0.19	73.26 ± 0.29	1270.48 ± 0.25	57.40 ± 0.17	49.32 ± 0.08
	Significance	0.773	0.913	**0.016**	0.893	0.699
AST	M ± SD	112.31 ± 0.21	193.48 ± 0.25	635.65 ± 0.32	68.68 ± 0.22	108.70 ± 0.25
	Significance	0.522	0.135	**0.018**	0.949	0.934
Urea	M ± SD	18.72 ± 0.24	26.13 ± 0.21	49.28 ± 0.25	27.67 ± 0.24	41.73 ± 0.2
	Significance	0.203	0.237	0.151	0.446	0.397
Creatinine	M ± SD	0.52 ± 0.01	0.63 ± 0.008	1.91 ± 0.009	0.58 ± 0.01	0.85 ± 0.07
	Significance	0.054	0.068	**0.028**	0.415	0.741
Glucose	M ± SD	84.70 ± 0.23	103.66 ± 0.3	101.26 ± 0.29	90.65 ± 0.22	49.67 ± 0.24
	Significance	0.214	0.169	0.441	0.87	0.922
*Hematological analysis*						
HB	M ± SD	12.48 ± 0.39	13.05 ± 0.32	10.04 ± 4.37	12.67 ± 0.24	12.20 ± 0.22
	Significance	0.894	0.694	**0.017**	0.579	0.437
PLT	M ± SD	420.65 ± 0.26	473.84 ± 0.32	1050.63 ± 0.34	461.65 ± 0.28	476.57 ± 0.31
	Significance	0.759	0.219	0.078	0.32	0.923
RBC	M ± SD	8.55 ± 0.021	9.94 ± 0.11	7.42 ± 0.18	9.28 ± 0.03	8.10 ± 0.21
	Significance	0.918	0.937	0.465	0.762	0.448
WBC	M ± SD	4.00 ± 0.051	8.92 ± 0.26	13.26 ± 0.13	4.28 ± 0.03	8.95 ± 0.05
	Significance	0.291	0.129	**0.0026**	0.522	0.484

Values are expressed as mean (M ±) and SD (Standard Deviation) with significant at *p* < 0.05

**Table 4:** Biochemical and hematological analysis in the main experiment (30 days)

Test	Groups	A	B3	C3	D1	E
*Biochemical analysis*						
ALT	M ± SD	55.89 ±0.18	73.26 ± 0.29	1350.48 ± 0.25	59.26 ± 0.02	41.70 ± 0.24
	Significance	0.934	0.913	**0.035**	0.554	0.402
AST	M ± SD	130.66 ± 0.3	193.48 ± 0.25	549.65 ± 0.30	138.03 ± 0.07	119.81 ± 0.978
	Significance	0.831	0.135	**0.018**	0.839	0.978
Urea	M ± SD	14.81 ± 0.181	21.13 ± 0.21	59.34 ± 0.25	19.38 ± 0.21	53.51 ± 0.22
	Significance	0.611	0.298	0.658	0.119	0.225
Creatinine	M ± SD	0.42 ± 0.008	0.54 ± 0.008	1.30 ± 0.009	0.47 ± 0.008	0.93 ± 0.07
	Significance	0.33	0.059	**0.013**	0.235	0.868
Glucose	M ± SD	71.94 ± 0.045	103.66 ± 0.3	98.37 ± 0.29	76.41 ± 0.17	62.76 ± 0.22
	Significance	0.912	0.169	0.341	0.715	0.951
*Hematological analysis*						
HB	M ± SD	13.75 ± 0.21	14.55 ± 0.32	9.35 ± 4.37	14.49 ± 0.2	15.20 ± 0.09
	Significance	0.959	0.694	**0.034**	0.942	0.961
PLT	M ± SD	403.95 ± 0.064	453.84 ± 0.32	1405.63 ± 0.34	436.65 ± 0.32	553.67 ± 0.27
	Significance	0.987	0.21	**0.028**	0.985	0.972
RBC	M ± SD	9.13 ± 0.03	9.94 ± 0.11	7.25 ± 0.18	9.34 ± 0.01	9.59 ± 0.09
	Significance	0.818	0.937	0.203	0.773	0.965
WBC	M ± SD	3.85 ± 0.022	8.92 ± 0.26	19.96 ± 0.13	4.15 ± 0.04	5.63 ± 0.03
	Significance	0.942	0.129	**0.005**	0.763	0.931

Values are expressed as mean (M ±) and SD (Standard Deviation) with significant at *p* <0.05

### Histopathological Studies

**Fig. 1:**
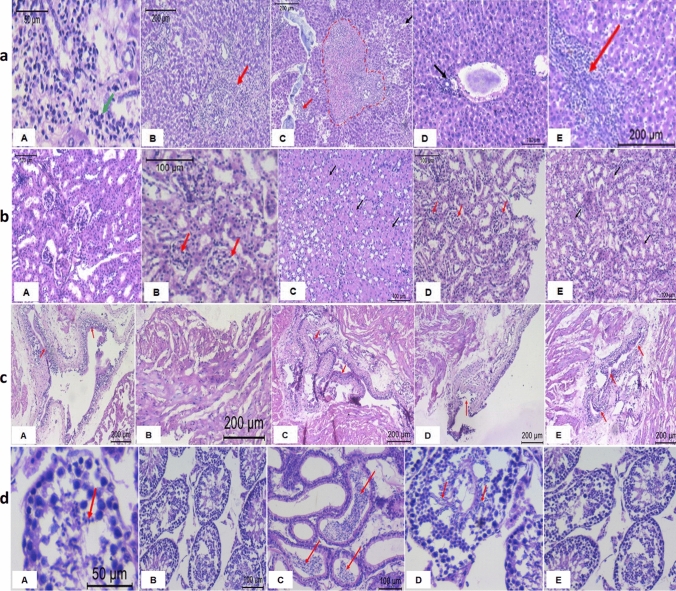
**a**, **b**, **c** and **d** Histological examinations of liver (**a**), kidney (**b**), urinary bladder (**c**) and testis (**d**) tissues of studied groups (A, B3, C3, D1 and E). Where, A is control group, B is group B3, C is group C3, D is group D1 and E is group E

Histopathological examination of liver, kidney, urinary bladder and testis in initial experiment (groups A, B3 and C3) and main experiment (groups D1 and E/30 days). It should be noted that in initial experiment and according to the biochemical and hematological analysis, 4 g was the best concentration of Artemisia extract in group B3, while 45 mg of the Actos drug was the best concentration in group C3. The results of the histopathological examination of the liver tissues in initial and main experiment (groups A, B3, C3, D1 and E) showed that a portal tract inflammation (green arrow) in control group (A), as shown in Fig. 1a. In group B3, there is a portal tract expansion by inflammatory cells (red arrow). In C3 group, there is focus of confluent necrosis (dotted lines), dilated sinusoids (black arrow) and portal tracts are expanded with inflammatory infiltrates (red arrow). In D1 group, the result showed mild portal tract inflammation (black arrow). Lastly, severe inflammation of the portal tract (red arrow) was shown in E group. In kidney tissues, the results showed that normal glomeruli, tubules and interstitium in control group (A). In both B3 and D1, there are patent glomeruli (red arrows), normal renal tubules and normal interstitium (Fig. 1b). In group C3, there is severe cloudy swelling of the renal tubules (black arrows). Mild cloudy swelling and tubular casts (black arrows) was shown in E group. For urinary bladder tissues, the results showed moderate inflammatory infiltrates in the submucosa (red arrows) in control group (A). In groups B3 and D1 (red arrow), there aremild inflammatory infiltrates in the submucosa, as shown in Fig. 1c. In C3 group, there are moderate to severe dysplastic changes in the lining urothelium and moderate inflammatory infiltrates in the submucosa (red arrows). In E group, moderate nonspecific inflammatory cellular infiltrates in the submucosa was found (red arrows). In testis tissues, the results showed normal spermatic tubules with active spermatogenesis reaching spermatozoa stage (red arrows) in both A (control) and D1 groups (Fig. 1d). In groups B3 and E, there are normal sized spermatic tubules showing normal active spermatogenesis. In group C3, there is normal epididymal tubules filled with spermatozoa (red arrows).

### Quantitative Real-Time PCR for TNF-α Gene

**Fig. 2:**
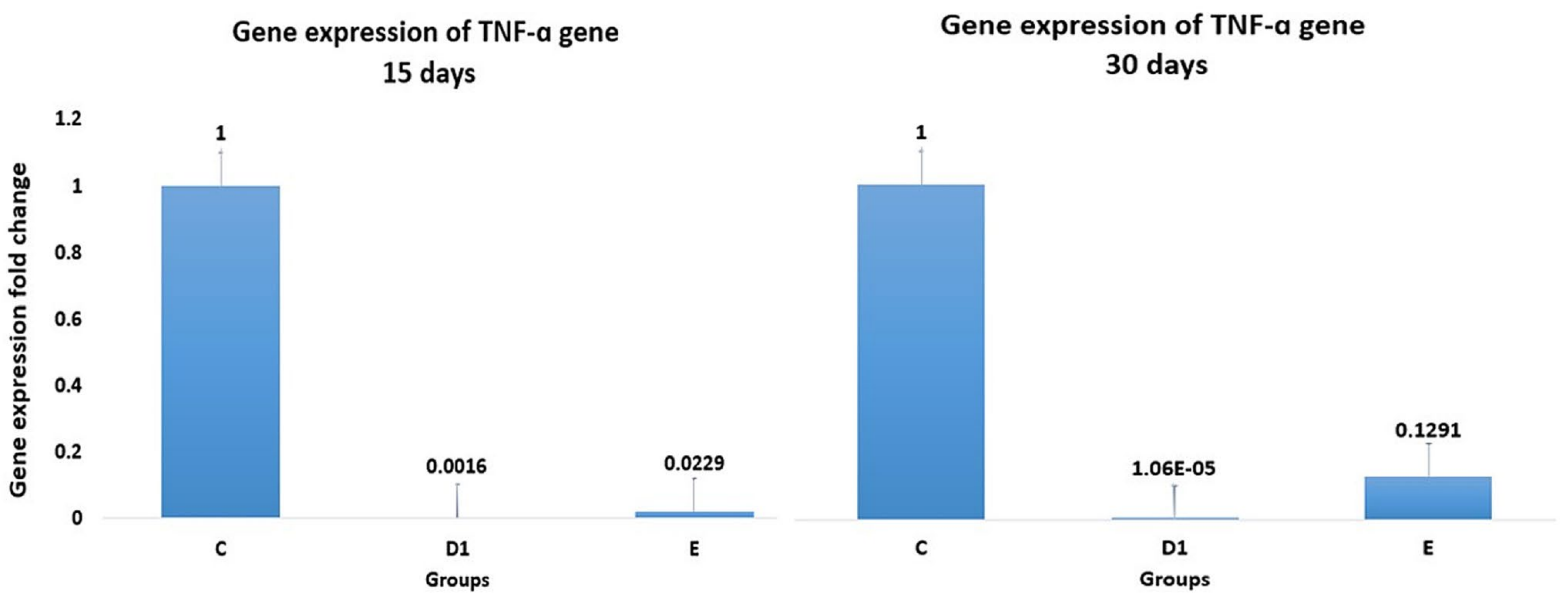
Gene expression level of TNF-*ɑ* gene in urinary bladder tissues of studied groups in the main experiment (15 and 30 days). Where, A is control group, D1 (Actos/Artemisia) and E (Actos/Artemisia/Ferric). The values are expressed as mean ± SD (Standard Deviation) with significant at *p* < 0.05

In 15 days of the main experiment, the TNF-ɑ oncogene expression levels of the urinary bladder tissues in D1 (45 mg/4 g of Actos/Artemisia) and E (45 mg/4 g/0.1 ml of Actos/Artemisia/Ferric) groups were significantly decreased by about 99.84% and 97.71%, respectively compared to the control group (A). In 30 days of the same main experiment, the expression level in D1 and E groups were significantly decreased by about 99.99% and 87.09%, respectively compared to the control group (A), as shown in Fig. 2.

## Discussion

The main objective of this study was to examine the activity of *Artemisia annua* L. extract as an anti-cancer and anti-inflammatory against the side effects of Actos drug (diabetic drug). Where, the biochemical results revealed that ALT, AST and Creatinine activities were significantly increased in group C3 compared to the control group, while they were significantly decreased in B3, D1 and E groups. For urea and glucose levels, the results showed non-significant differences in all the groups compared to the control group. In related previous studies, [19] has found no changes in the liver (ALT and AST) and kidney (urea and Creatinine) functions in all the EAA-treated (*Artemisia Annua* Ethanol extract) groups compared to the control group and the author recommended that EAA treatment may not predispose the users of this plant to hepatotoxicity and renal dysfunction. Concerning glucose, different types of AA extracts were used by [20] to improve insulin resistance through reduction of TNF-alpha. These previous results confirm our findings that higher levels of bioactive components might be present in the higher concentrations of Artemisia extracts. Hence, the Artemisia extract can be used as a treatment against the negative side effects of Actos which used as a diabetic drug.

The hematological results revealed that HB count was significantly decreased in group C3, but PLT and WBC counts were significantly increased, while, they had no effect in B3, D1 and E groups. On the other hand, RBC count showed non-significant differences in B3, C3, D1 and E groups compared to the control group. In a previous related study to [19], there was no any significant change in the counts of HB, RBC and PLT after EAA treatment in rats. The author has also found that the different doses of EAA treatment did not cause haematotoxicity. It should be noted that, these findings agree with our results of HB, RBC and PLT counts, while do not agree with the result of WBC count.

The results of the histopathological examinations of liver tissues showed that the Actos drug (C3 group) causes focus of confluent necrosis, dilated sinusoids and portal tracts which expanded with inflammatory infiltrates. In kidney tissues, severe cloudy swelling of the renal tubules was caused by Actos drug. For bladder tissues, the results revealed that the Actos drug causes moderate to severe dysplastic changes in the lining urothelium and moderate inflammatory infiltrates in the submucosa. In testis tissues, the results of Actos drug showed normal spermatic tubules with active spermatogenesis reaching spermatozoa stage in all treated and non-treated animal groups. In contrast, the results of *Artemisia annua* extract treatment (D1 and E groups) showed that decreasing of the previous harmful effects of the Actos drug in liver tissues. For the kidney tissues, the results of *Artemisia annua* L. extract treatment showed that marked histopathologic improvement. In urinary bladder tissues, the results of Artemisia annua extract treatment showed decreasing of the previous harmful effects of the Actos drug. In testis tissues, the results of *Artemisia annua* showed normal spermatic tubules with active spermatogenesis reaching spermatozoa stage in all treated and non-treated animal groups. In the previous studies, the histopathology indicating that the liver was not adversely affected by EAA further confirmed the earlier statements that EAA may not be toxic to the liver [19, 20]. While, [22] has found that the acute administration of large doses of pioglitazone (Actos drug) caused ventricular hypertrophy with congestion of liver in mice which can happen with accidental over dose of pioglitazone in patients. However, these previous findings are agreed and confirmed with our findings in the liver tissues. [21, 23] have found that therapy duration and cumulative dose of pioglitazone (Actos drug) increased the risk of developing chronic kidney disease. While, [20] has found that EAA may not be toxic to the kidney. In studies by [24, 25], sub-chronic use of pioglitazone (Actos drug) may lead to bladder affection, however, current evidence suggests that pioglitazone may increase the risk of bladder cancer, possibly in a dose‐ and time‐dependent manner. On the other hand, [26] has found that Artemisinin which isolated of *Artemisia annua* plant could induce autophagy dependent apoptosis through AMPK-mTOR-ULK1 axis in the bladder cancer cells. Consequently, the Artemisinin holds promising future as a novel medicine or an adjuvant strategy for the treatment of bladder cancer. In a study to [19], the author has found that the testis were not adversely affected by EAA further confirmed their earlier statements that EAA may not be toxic to the testis in rats. While, [27] has found that the protective effect of pioglitazone (Actos drug) possibly involves the reduction of oxidative stress by decreasing inflammatory status by decreasing the expressions of pro-inflammatory cytokines TNF-*α* levels in rats. However, our findings agree and confirm the author’s previous results, where, the *Artemisia annua* L. extract and Actos drug have no negative effects on mice fertility.

The results of *Artemisia annua* L. extract treatment in the main experiment (15 and 30 days) showed that the TNF-ɑ oncogene expression levels in the bladder tissues were significantly decreased in groups D1 and E compared to the control group. In some previous study, [28] has found that the *Artemisia annua* extract was a potent inhibitor of TNF-*α* by activated neutrophils with a clear dose–response effect in rat. The author has found that the extract showed statistically significant inhibition of TNF-α production at all concentrations down to 2.5 µg/ml (24% inhibition). In another study on human cell lines, [14] has found that the *Artemisia annua* (AA) 90% ethanol Phyto-extract showed a greater ability to reduce TNF-α oncogene expression. [29] has isolated six new sesquiterpenoids from the aerial parts of *Artemisia annua*, three of these compounds showed inhibitory activities against the production of inflammatory cytokines TNF-α in lipopolysaccharide (LPS)-induced (murine macrophage cell line). Our findings agree and support the previous results, where the TNF-*ɑ* oncogene expression levels were significantly decreased by using *Artemisia annua* L. extract treatment.

## Conclusions

In conclusion, treatment with Actos drug alone in male albino mice was effective for type 2 diabetes, but with companion side effects. Treatment with Artemisia extract alone had no side effects on all different tissues. The concurrent treatment with both Actos drug and Artemisia extract was effective against the harmful side effects of Actos drug, moreover, this concurrent did not change the efficiency of the Actos drug as a diabetic drug. Consequently, it could be said that the *Artemisia annua* L. extract is a promising natural product against the side effects of Actos drug in mice.

## Data Availability

Data sharing not applicable to this article as no datasets were generated or analyzed during the current study.

## References

[1] Mizrachi E, Bernel-Mizrachi C, Cooper DH, Krainik AJ, Lubner SJ, Reno HEL (2007). Diabetes mellitus and related disorders. Washington manual of medical therapeutics.

[2] Desouza CV, Shivaswamy V (2010). Pioglitazone in the treatment of type 2 diabetes: Safety and efficacy review. Clinical Medicine Insights. Endocrinology and Diabetes.

[3] Belfort R, Harrison SA, Brown K, Darland C, Finch J, Hardies J, Balas B, Gastaldelli A, Tio F, Pulcini J, Berria R (2006). A placebo-controlled trial of pioglitazone in subjects with nonalcoholic steatohepatitis. The New England Journal of Medicine.

[4] Nolte MS, Katzung BG, Masters SB, Trevor AJ (2009). Pancreatic hormones and antidiabetic drugs. Basic and clinical pharmacology.

[5] Schernthaner G, Matthews DR, Charbonnel B, Hanefeld M, Brunetti P (2004). Quartet study group. Efficacy and safety of pioglitazone versus metformin in patients with type 2 diabetes mellitus: A doubleblind, randomized trial. Journal of Clinical Endocrinology and Metabolism.

[6] Patel C, Wyne KL, McGuire DK (2005). Thiazolidine-diones peripheral oedema and congestive heart failure: What is the evidence?. Diabetes & Vascular Disease Research.

[7] Shukla R, Karla S (2011). Pioglitazone: Indian perspec-tive. Indian Journal of Endocrinology and Metabolism.

[8] Saha SK, Chowdhury AA, Bachar SC, Das SC, Kuddus RH, Uddin MA (2013). Comparative in vitro-in vivo correlation analysis with pioglitazone tablets. Asian Pacific Journal of Tropical Disease.

[9] FDA Drug Safety Podcast (2022). Updated FDA review concludes that use of pioglitazone may be linked to an increased risk of bladder cancer (FDA Drug Safety Podcast: Updated FDA review concludes that use of pioglitazone may be linked to an increased risk of bladder cancer | FDA).

[10] Hsu E (2006). The history of Qinghao in the Chinese materia medica. Transactions of the Royal Society of Tropical Medicine and Hygiene.

[11] Ko YS, Lee WS, Panchanathan R, Joo YN, Choi YH, Kim GS, Jung JM, Ryu CH, Shin SC, Kim HJ (2016). Polyphenols from *Artemisia **annua* L. inhibit adhesion and EMT of highly metastatic breast cancer cells MDA-MB-231. Phytotherapy Research.

[12] Wang X, Lin Y (2008). Tumor necrosis factor and cancer, buddies or foes. Acta Pharmacologica Sinica.

[13] Jurisic V, Srdic T, Konjevic G, Bogdanovic G, Colic M (2011). TNF-*α* induced apoptosis is accompanied with rapid CD30 and slower CD45 shedding from K-562 cells. The Journal of membrane biology.

[14] Abate G, Zhang L, Pucci M, Morbini G, Mac Sweeney E, Maccarinelli G, Ribaudo G, Gianoncelli A, Uberti D, Memo M, Lucini L (2021). Phytochemical analysis and anti-inflammatory activity of different ethanolic phyto-extracts of *Artemisia **annua* L.. Biomolecules.

[15] Jurisic V (2020). Multiomic analysis of cytokines in immuno-oncology. Expert Review of Proteomics.

[16] Van der Kooy F, Verpoorte R (2011). The content of artemisinin in the *Artemisia **annua* tea infusion. Planta Medica.

[17] Chade DC, Borra RC, Nascimento IP, Villanova FE, Leite LC, Andrade E, Srougi M, Ramos KL, Andrade PM (2008). Immunomodulatory effects of recombinant BCG expressing pertussis toxin on TNF-alpha and IL-10 in a bladder cancer model. The Journal of Experimental & Clinical Cancer Research.

[18] Hideki K, Mitsuharu Y, Hidenori Z, Masayuki T, Isao A (2009). Sex differences in the expression profile of acid-sensing ion channels in the mouse urinary bladder: A possible involvement in irritative bladder symptoms. BJU International.

[19] Eteng MU, Abolaji AO, Ebong PE, Brisibe EA, Dar A, Kabir N, Iqbal Choudhary M (2013). Biochemical and haematological evaluation of repeated dose exposure of male Wistar rats to an ethanolic extract of *Artemisia **annua*. Phytotherapy Research.

[20] Ghanbari M, Sadeghimahalli F (2022). Aqueous and alcoholic extracts of *Artemisia **annua* L. improved insulin resistance via decreasing TNF-alpha, IL-6 and free fatty acids in high-fat diet/streptozotocin-induced diabetic mice. Avicenna Journal of Phytomedicine.

[21] Alsulami MN, Mohamed YM (2022). Therapeutic efficacy of *Artemisia **annua* ethanol extract versus albendazole on migrating & encysted *Trichinella* spiralis larvae in mice. Journal of The Egyptian Society Parasitology.

[22] Chinnam P, Mohsin M, Shafe LM (2012). Evaluation of acute toxicity of pioglitazone in mice. Toxicology International.

[23] Lee MY, Hsiao PJ, Yang YH, Lin KD, Shin SJ (2014). The association of pioglitazone and urinary tract disease in type 2 diabetic Taiwanese: Bladder cancer and chronic kidney disease. PLoS ONE.

[24] Elshama S, El-Kenawy A, Osman HE (2016). Toxicological evaluation of sub-chronic use of pioglitazone in mice. Iranian Journal of Basic Medical Sciences.

[25] Wang JX, Tang W, Zhou R, ShiL PW, Zhang Y (2008). The new water-soluble artemisinin derivative SM905 ameliorates collagen-induced arthritis by suppression of inflammatory and Th17 responses. British journal of pharmacology.

[26] Zhou X, Chen Y, Wang F, Wu H, Zhang Y, Liu J, Cai Y, Huang S, He N, Hu Z, Jin X (2020). Artesunate induces autophagy dependent apoptosis through upregulating ROS and activating AMPK-mTOR-ULK1 axis in human bladder cancer cells. Chemico-Biological Interactions.

[27] Malekifard F, Dalirezh N, Soleimanzadeh A (2018). Modulatory effect of pioglitazone on sperm parameters and oxidative stress, apoptotic and inflammatory biomarkers in testes of streptozotocin-induced diabetic rats. International Journal of Machine Learning.

[28] Hunt S, Yoshida M, Davis EJ, Greenhill NS, Davis PF (2015). An extract of the medicinal plant *Artemisia **annua* modulates production of inflammatory markers in activated neutrophils. Journal of Inflammation Research.

[29] Qin DP, Li T, Shao JR, He XQ, Shi DF, Wang ZZ, Xiao W, Yao XS, Li HB, Yu Y (2021). Arteannoides U–Z: Six undescribed sesquiterpenoids with anti-inflammatory activities from the aerial parts of *Artemisia **annua* (Qinghao). Fitoterapia.

